# MiR-378b Modulates *Chlamydia*-Induced Upper Genital Tract Pathology

**DOI:** 10.3390/pathogens10050566

**Published:** 2021-05-07

**Authors:** Stephanie R. Lundy, Kobe Abney, Debra Ellerson, Joseph U. Igietseme, Darin Carroll, Francis O. Eko, Yusuf O. Omosun

**Affiliations:** 1 Department of Microbiology, Biochemistry & Immunology, Morehouse School of Medicine, Atlanta, GA 30310, USA; slundy@msm.edu (S.R.L.); kabney1@spelman.edu (K.A.); jigietseme@cdc.gov (J.U.I.); feko@msm.edu (F.O.E.); 2 Department of Chemistry and Biochemistry, Spelman College, Atlanta, GA 30310, USA; 3 Centers for Disease Control & Prevention (CDC), Atlanta, GA 30333, USA; dellerson@cdc.gov (D.E.); dcarroll@cdc.gov (D.C.)

**Keywords:** miR-378b^−/−^ mice, Chlamydial pathogenesis, *Chlamydia muridarum*, infertility

## Abstract

Genital *Chlamydia trachomatis* infection causes severe reproductive pathologies such as salpingitis and pelvic inflammatory disease that can lead to tubal factor infertility. MicroRNAs (miRNAs) are evolutionarily conserved regulators of mammalian gene expression in development, immunity and pathophysiologic processes during inflammation and infection, including *Chlamydia* infection. Among the miRNAs involved in regulating host responses and pathologic outcome of *Chlamydia* infection, we have shown that miR-378b was significantly differentially expressed during primary infection and reinfection. In this study, we tested the hypothesis that miR-378b is involved in the pathological outcome of *Chlamydia* infection. We developed miR-378b knockout mice (miR-378b^−/−^) using Crispr/Cas and infected them along with their wild-type (WT) control with *Chlamydia* to compare the infectivity and reproductive pathologies. The results showed that miR-378b^−/−^ mice were unable to clear the infection compared to WT mice; also, miR-378b^−/−^ mice exhibited a relatively higher *Chlamydia* burden throughout the duration of infection. However, gross pathology results showed that miR-378b^−/−^ mice had significantly reduced uterine dilatations and pathologic lesions after two infections compared to WT mice. In addition, the pregnancy and fertility rates for infected miR-378b^−/−^ mice showed protection from *Chlamydia*-induced infertility with fertility rate that was comparable to uninfected WT mice. These results are intriguing as they suggest that miR-378b is important in regulating host immune responses that control Chlamydial replication and drive the inflammation that causes complications such as infertility. The finding has important implications for biomarkers of Chlamydial complications and targets for prevention of disease.

## 1. Introduction

Genital infection by the Gram negative, obligate intracellular bacterium, *Chlamydia trachomatis*, is the most common bacterial sexually transmitted disease worldwide and therefore of high public health concern [1,2]. There are an estimated 130 million cases of genital *Chlamydia* infections worldwide, with an estimated 4 million new cases occurring annually in the United States alone [3]. However, many cases are unreported due to their asymptomatic nature [1,2]. Any sexually active individual is at risk of *Chlamydia* infection; however, two-thirds of new infections occur among young women between 15 and 24 [1,4]. In women, *C. trachomatis* infects the columnar epithelial cells of the cervix and urethra, causing uncomplicated cervitis and urethritis with symptoms including urinary tract infection, vaginal discharge, and bleeding [1,2]. *Chlamydia* migrates into the upper genital tract leading to disease complications in the uterus (endometritis) and fallopian tubes (salpingitis) [1,2]. *Chlamydia* causes symptomatic and asymptomatic pelvic inflammatory disease (PID) experienced by 2–5% of women with an untreated infection. PID is known to cause irreversible damage to the uterus, fallopian tubes, and surrounding tissues leading to pelvic pain, tubal factor infertility, and potentially fatal ectopic pregnancy [1,2].

Several host and pathogen factors contribute to *Chlamydia*-associated pathology, including the host immune response to infection. However, it is still unclear which molecular mechanisms produce these complications in the female genital tract. MicroRNAs (miRNA) have been implicated in the development of adverse tissue pathologies following genital Chlamydial infection [5,6,7,8,9]. miRNAs are endogenous, short, non-coding RNAs. RNA Polymerase II transcribes miRNAs to form the primary miRNA [10,11]. RNaseA’ III-type endonucleases, Drosha and Dicer, process the primary miRNA in the nucleus and cytosol, respectively, to form mature miRNA duplex [10,11]. A single strand of the miRNA is loaded onto an AGO protein creating an RNA-induced silencing complex (RISC) that can target mRNA, inducing translational repression affecting several pathways [10,11].

We and others have previously reported that *Chlamydia*-infected mice had differentially expressed miRNAs [5,9,12]. By using deep RNA sequencing in the murine model of genital Chlamydial infection that exhibited key infection and pathologic features of the human disease, we identified significantly more differentially expressed miRNAs by analyzing the expression of all annotated miRNAs following the infections [5]. Our study identified miR-378b that was upregulated the first week after infection and downregulated eight weeks after a primary infection and reinfection, and it was also the most differentially expressed miRNA overall [5]. The finding suggested miR-378b expression may play a key role in Chlamydial pathogenesis. miR-378b is a member of the miR-378b family that includes miR-378a-3p, miR-378b, miR-378c, miR-378d, and miR-378e. In humans, it is located on chromosome three. Previous studies show that miR-378b is expressed in many tissues [5,13,14,15]. The role of miR-378 on inflammation and fibrosis has been conflicting. Some studies have shown that miR-378 was pro-fibrotic and pro-inflammation [15]. However, other studies showed that overexpression of miR-378 is associated with reducing fibrosis, hypertrophy, and tubular fibrosis, attenuating ischemic injury, and inhibiting epithelial cell proliferation, migration, and differentiation [13,16,17,18]. Additional studies also showed that overexpression of miR-378 is associated with reduced liver fibrosis [18], reduced mesangial hypertrophy, and kidney tubular fibrosis [16], attenuating ischemic injury [16], inhibiting proliferation, migration, and promoting differentiation in keratinocytes [14]. Moreover, we recently showed that miR-378a was down regulated after vaccination with the *Vibrio cholerae* ghost vaccine against *Chlamydia* [19]. Because miR-378b was prominently differentially expressed during genital Chlamydial infection leading to infertility, its function in *Chlamydia* infectivity and associated tissue pathology was investigated.

Moreover, while several studies have shown that *Chlamydia* infection causes the differential expression of several miRNAs in the female genital tract [5,6,7,9,12], it is unclear if any of these miRNAs are linked to *Chlamydia* infectivity and development of tissue pathology. We hypothesize that miR-378b is essential for regulating the host inflammatory and pathologic responses leading to *Chlamydia* complications such as Tubal Factor Infertility (TFI). We will associate the expression of miR-378b to infertility caused by *Chlamydia* infection and show that it is a central player in the process of Chlamydial pathogenesis leading to TFI. We use a C57Bl/6J miR-378b^−/−^ mouse model of Chlamydial genital infection to determine the effect of miR-378b in *Chlamydia* pathogenesis. We determine the Chlamydial infectivity, gross pathology of the genital tract after *Chlamydia* infection, cytokine secretion in the genital tract and perform fertility assay in infected miR-378b^−/−^ mice. Our results show that, in the absence of miR-378b, the *Chlamydia* burden was high but the deleterious pathology associated with the infection was reduced and the infertility was prevented.

## 2. Results

### 2.1. Effect of miR-378b on Chlamydia Infectivity

**Figure 1:**
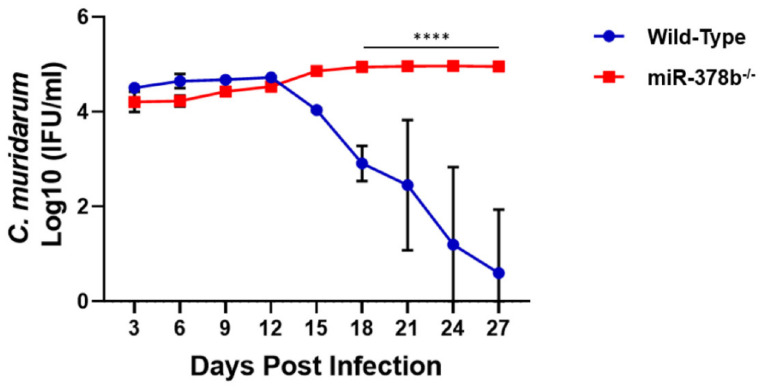
Effect of miR-378b on *Chlamydia* infectivity. Female wild-type and miR-378b^−/−^ mice were infected with *C. muridarum*. Infectivity was determined up to 27 days post-infection. Data were analyzed using two-way repeat measure ANOVA and a Tukey post hoc test (**** *p* < 0.0001).

Female wild-type and miR-378b^−/−^ mice were intravaginally infected with 1 × 10^5^ Infectious Units (IFUs) *Chlamydia muridarum* (*C. muridarum*). miR-378b^−/−^ mice had a significantly higher bacterial load from day 18 to 27 post-infection (*p* < 0.0001) than wild-type infected mice. miR-378b^−/−^ mice were also unable to clear the infection (Figure 1). This result suggests that miR-378b may be necessary for the clearance of *Chlamydia* infection.

### 2.2. Effects of miR-378b on the Female Genital Tract Pathology after Chlamydia Infection

**Figure 2:**
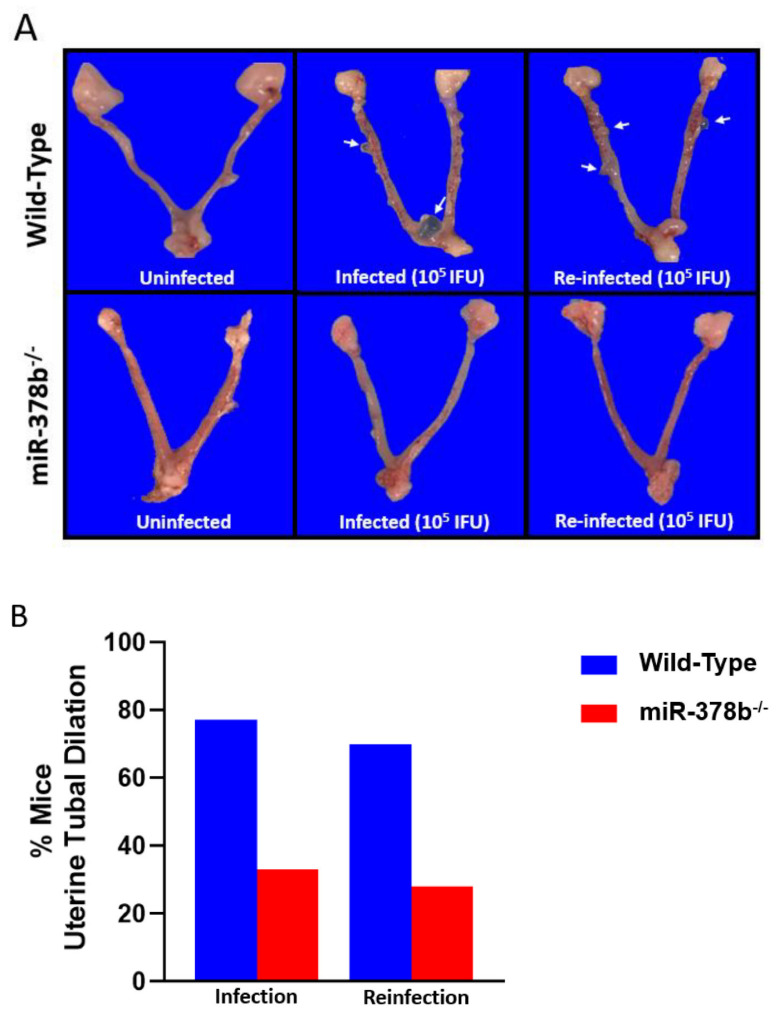
Effect of miR-378b on gross pathology after *Chlamydia* infection. Wild-type (n = 19) and miR-378b^−/−^ (n = 13) female mice were infected and reinfected with 1 × 10^5^ *C. muridarum*. (**A**) Wild-type infected mice had more uterine tubal dilation compared to miR-378b^−/−^ mice that had minimal uterine tubal pathology. (**B**) Percentage of wild-type and miR-378b^−/−^ mice with uterine tubal dilation. The white arrows indicate uterine dilations.

Genital tract tissues excised from female wild-type and miR-378b^−/−^ mice infected with 1 × 10^5^ IFUs *C. muridarum* were evaluated for pathological lesions. Following infection, wild-type mice had more uterine tubal dilations and lesions, while miR-378b^−/−^ mice had minimal uterine pathology. Similar results were obtained following reinfection of mice with 1 × 10^5^ IFU *C. muridarum* (Figure 2A) and infection with 1 × 10^6^ IFU *C. muridarum* (Figure S3), suggesting that a loss of miR-378b may have a protective role against the development of genital tract pathology following *Chlamydia* infection.

### 2.3. Effect of miR-378b on Cytokine and Chemokine Secretion

**Figure 3:**
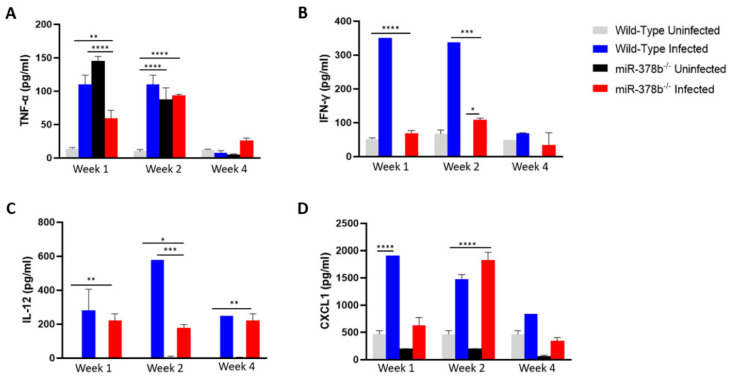
Effect of miR-378b on cytokine secretion after *Chlamydia* infection. Cytokine concentrations in vaginal lavages collected from wild-type and miR-378b^−/−^ mice were determined. (**A**) TNF-α. (**B**) IFN-γ (**C**) IL-12 (**D**) CXCL1. The data was analyzed using a one-way ANOVA and Tukey post hoc test (* *p* < 0.05; ** *p* < 0.01; *** *p* < 0.001; **** *p* < 0.0001).

To determine the effect of miR-378b on immune response following *C. muridarum* infection, vaginal lavage was collected weekly from uninfected and infected wild-type and miR-378b^−/−^ mice and assayed for antigen-specific cytokine secretion. TNF-α, IFN-γ and IL-12 secretion were significantly lower in miR-378b^−/−^ mice than wild-type mice at different time points after infection (Figure 2A–C), except for TNF-α four weeks post infection. CXCL1 secretion was significantly lower in miR-378b^−/−^ at week 1 (*p* < 0.0001), significantly higher in week 2 (*p* < 0.0001), and significantly lower in week 4 (*p* < 0.05) compared to wild-type infected mice (Figure 3D). This data suggests that the deletion of miR-378b dampens the pro-inflammatory immune response observed following a *Chlamydia* infection. This might be the reason for both the high infectivity and the reduced gross pathology.

### 2.4. Effect of miR-378b on Female Mouse Fertility after Chlamydia Infection

**Figure 4:**
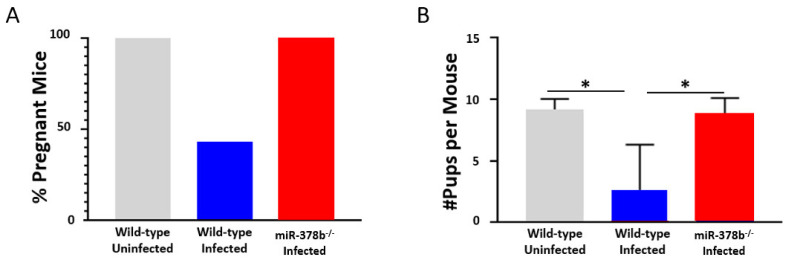
Effect of miR-378b on fertility after *Chlamydia* infection. The fertility of wild-type (n = 10, 5 uninfected, 5 infected) and miR-378b^−/−^ (n = 6) mice were measured using a fertility assay after infection with *C. muridarum*. (**A**) miR-378b^−/−^ mice had a 100% rate of pregnancy compared to the 50% rate of pregnancy in wild-type infected mice. (**B**) The fertility rate was significantly higher in infected miR-378b^−/−^ mice and was comparable to uninfected wild-type mice. The fertility rate was determined by analyzing the number of pups per mouse. The data were analyzed using a one-way ANOVA and Tukey post hoc test (* *p* < 0.05).

We determined the effect of miR-378b on the fertility rate of *Chlamydia*-infected mice by evaluating the pregnancy rate and mean number of embryos in infected wild-type and miR-378b^−/−^ mice. Infected miR-378b^−/−^ mice had a 100% pregnancy rate while 50% of infected wild-type mice became pregnant (Figure 4A). Infected miR-378b^−/−^ mice had significantly more pups per mouse than infected wild-type mice (Figure 4B).

## 3. Discussion

*Chlamydia trachomatis* is associated with several adverse pathologies in women, such as PID and salpingitis leading to tubal factor infertility [1]. MiRNAs have been shown to be associated with *Chlamydia* pathogenesis and immune response in several studies [5,6,7,9,12,19]. However, no studies have offered a direct association between a specific miRNA and *Chlamydia* pathogenesis. In our previous study [5], we identified miR-378b as the most differentially expressed miRNA in the reproductive system after genital *Chlamydia* infection [5]. The finding suggested miR-378b expression may play a key role in Chlamydial pathogenesis. Some studies have shown that miR-378 was pro-fibrotic and pro-inflammation [15], while other studies have shown an anti-fibrotic and anti-inflammatory effect [16,18,20]. This study analyzed miR-378b knockout (miR-378b^−/−^) mice that were generated using the CRISPR/CAS technique and showed a direct association between miR-378b and *Chlamydia* pathogenesis.

To determine the influence of miR-378b on *Chlamydia* infectivity, we infected miR-378b^−/−^ mice with 1 × 10^5^ *C. muridarum* and compared the infectivity and reproductive system pathologies to wild-type (WT) mice. We hypothesized that the absence of miR-378b would be associated with increased infectivity because of the role of miR-378 in host immune and inflammatory responses. The results showed that miR-378b^−/−^ mice could not clear their *Chlamydia* infection, with a prolonged *Chlamydia* burden that lasted over 27 days of infection, while the control WT mice resolved their infection by day 27. Although we had predicted greater pathologies for the infected miR-378b^−/−^ than infected WT mice, due to the high *Chlamydia* infectivity, the results revealed that the infected miR-378b^−/−^ mice developed reduced uterine dilatations and pathologic lesions. Furthermore, in fertility studies, the infected miR-378b^−/−^ mice were protected from *Chlamydia*-induced infertility with a pregnancy and fertility rate comparable to uninfected WT mice. These results were impressive as they show that the absence of miR-378b protected mice from the pathologic consequences of genital *Chlamydia* infection including infertility.

To investigate the host immune response mechanisms underlying the protection of miR-378b^−/−^ mice from reproductive system pathologies and infertility while accommodating microbial infectivity, we analyzed cytokines in the vaginal lavage of these mice. The results showed that the pro-inflammatory IL-12, IFN-γ and TNF-α levels in the vaginal secretions were lower in infected miR-378b^−/−^ mice compared to infected WT mice. Moreover, there was a reduction in CXCL1 secretion, a key chemokine for neutrophils, after the first week of infection in infected miR-378b^−/−^ mice. The reduction in vaginal mucosal pro-inflammatory cytokines, that have been established to play a role in *Chlamydia* clearance, may explain the high genital *Chlamydia* burden in miR-378b^−/−^ mice. However, the reduced pro-inflammatory cytokines resulted in the moderate reproductive pathology and protection from infertility observed in these miR-378b^−/−^ mice, since excessively high levels of pro-inflammatory cytokines are associated with reproductive system pathologies associated with *Chlamydia* [1,21].

Acute neutrophil (PMN) recruitment has been associated with the reproductive system pathologies caused by genital *Chlamydia* infection [22]. CXCL1 is responsible for the recruitment of neutrophils to the sites of infection [23,24]. Therefore, the significantly reduced secretion of CXCL1 in the first and fourth weeks of infection of miR-378b^−/−^ mice may imply a reduced number of neutrophils recruited to the site of infection of miR-378b^−/−^ mice compared to infected WT mice, hence the reduced pathologies in the former.

The findings in this study corroborate previous studies showing that mice deficient of the pro-inflammatory CCR5 chemokine receptor were fertile, although they had a relatively high *Chlamydia* burden after a genital infection [25]. The question that requires answering is what genes are being regulated by miR-378b that are leading to these outcomes. While it is unclear whether CCR5, its chemokine ligands or CXCL1 are the target of miR-378b, the determination of which genes are differentially expressed in the upper genital tract of infected miR-378b^−/−^ mice will enable us to discover the genes that can be targeted in novel therapy to treat complications arising from *Chlamydia* infection. We intend to analyze these miR-378b associated genes to understand their role in these outcomes. For instance, miR-378b is associated with liver and kidney fibrosis [15,16,18] and regulates hedgehog cell growth and differentiation [18]. Abnormal hedgehog signaling has been reported in many cancer types, including basal cell cancer, prostate, mammary glands, and lungs [26,27].

The results from this study suggest that the deficiency of miR-378b has a beneficial role in mitigating the development of *Chlamydia*-associated pathology in the female genital tract and preventing the development of infertility in female mice. These data also suggest that anti-miR-378b inhibitors or small molecules could be used as possible therapeutics in women infected with *Chlamydia* to prevent the development of adverse pathology and infertility. This study is the first to associate outcomes of *Chlamydia* pathogenesis with a specific miRNA.

## 4. Materials and Methods

### 4.1. Animal Protocol Approval Statement

This study was carried out in strict adherence to the recommendations in the Guide for the Care and Use of Laboratory Animals of the National Institutes of Health. The Institutional Animal Care and Use Committee (IACUC) of Morehouse School of Medicine approved the study protocol (Protocol Number: 16–24).

### 4.2. Animals

#### 4.2.1. Generation of C57BL/6J miR-378b^−/−^ mice

We recently bred miR-378b^−/−^ mice generated using CRISPR/CAS, with the assistance of GenEdits, a genetic engineering company resident in Morehouse School of Medicine (Figure S1). These mice are reproductively viable, and we have used them in the experiments in this study (Figures S1 and S2).

#### 4.2.2. Light Conditions

Female C57BL/6J mice (n = 19) (Jackson Laboratory, Bar Harbor, MA, USA) and C57BL/6J miR-378b^−/−^ (n = 19) mice were housed under normal light (LD) conditions of 12 h lights on and 12 h lights off.

### 4.3. Chlamydia muridarum Stock

*Chlamydia muridarum niggs (C. muridarum)* stocks, purified using renografin and centrifugation, (Centers for Disease Control, Atlanta, GA, USA), were diluted in sterile Sucrose Phosphate Glutamate (SPG) transport media to a final concentration of 1 × 10^5^ or 1 × 10^6^ Infectious Units (IFUs).

### 4.4. C. muridarum Infectivity Assay

All mice received a subcutaneous injection of Depo Provera (2.5 mg/mouse), medroxyprogesterone acetate (Pfizer, New York, NY, USA) in sterile Phosphate Buffer Saline (PBS) to synchronize the estrous cycle. Mice were anesthetized using isoflurane and were either sham infected (20 µL SPG) or intravaginally infected seven days later at six weeks old with 1 × 10^5^ or 1 × 10^6^ IFU *C. muridarum* at 10:00 AM (n = 12). *Chlamydia*-infected mice were swabbed every three days for 27 days, and the bacteria were isolated and cultured to follow the progression and clearance of the infection. For *Chlamydia* reinfection, the procedure stated above was repeated 30 days after *Chlamydia* infection. Swabs were stored in conical tubes containing glass beads in SPG buffer.

### 4.5. C. muridarum Isolation from Vaginal Swabs

McCoy cells (concentration 1 × 10^5^) were plated in a 24-well plate. Conical tubes containing vaginal swabs and glass beads in SPG buffer were vortexed for thirty seconds. The swab was removed, and contents of conical tubes were sonicated for twenty seconds, then vortexed again for 10 s. Inoculum (300 µL) was transferred into each well. The plates were centrifuged for one hour at 2200 rpm, then incubated at 37 °C for one hour. The supernatant was removed, and 1 mL of warm antibiotic-free Iscove’s media containing 10% FBS and 1.5 mg/mL cycloheximide was added to each well. The plate was incubated at 37 °C for 48 h.

### 4.6. C. muridarum Staining

Media was removed from 24-well plates and washed twice with PBS. Cells were fixed with ice-cold 100% methanol (1 mL) for one hour. Methanol was removed, and one drop of *Chlamydia* Pathfinder (Bio-Rad Laboratories Inc., Hercules, CA, USA) was added, and the plate was incubated in the dark for one hour. The *Chlamydia* Pathfinder was removed, and the plates were washed twice with distilled water. *Chlamydia* inclusions were counted using a fluorescence microscope.

### 4.7. Pathology

Chlamydia-associated genital tract pathology in wild-type and miR-378b^−/−^ was investigated. Mice were euthanized by carbon dioxide and cervical dislocation 34 days post-infection. Following euthanasia, the genital tract was dissected, and the number of paraovarian cysts and tubal dilations was counted.

### 4.8. Vaginal Lavage

After infection, vaginal lavage was collected once a week for four weeks. A pipette tip containing sterile PBS (60 µL) was inserted into the vagina of each mouse. The PBS was dispensed and drawn up five times then transferred into an Eppendorf tube. The lavage was placed on dry ice following collection and stored at −80 °C.

### 4.9. Cytokine Assay

Cytokine concentrations (C-X-C motif ligand (CXCL)-1, Tumor Necrosis Factor (TNF)-α, Interferon (IFN)-γ, and IL-12) in the collected vaginal lavage was determined using the R&D Systems Magnetic Luminex Assay, Mouse Premixed Multi-Analyte Kit (R&D, Hercules, CA, USA) following the manufacturer’s protocol. The concentration of cytokine or chemokine in each sample was calculated by extrapolation from a standard calibration curve. The mean and standard deviation for replicate samples were calculated.

### 4.10. Fertility Assay

Five weeks post-infection, wild-type uninfected (n = 5) and wild-type (n = 5) infected mice and miR-378b^−/−^ (n = 6) infected mice were placed in cages with fertile, proven male C57Bl/6J mice (Jackson Laboratory, Bar Harbor, MA), at a ratio of two females to one male mouse. One week after the addition of male mice, the female mice were weighed every three days until they gained approximately 10 g to confirm pregnancy. After the pregnancy was confirmed by weight gain, mice were euthanized and dissected to determine the number of pups [28].

### 4.11. Statistical Analysis

Statistical differences in immune response and fertility rate between infected and non-infected mice were analyzed using a one-way analysis of variance (ANOVA). The difference in infectivity between the treatment groups was determined using a two-way ANOVA. A post hoc test was performed to determine the treatment groups’ actual statistical relationship following the one or two-way ANOVA. A Chi-square test was used to determine the difference in pregnancy rate between the different treatment and mouse groups. Statistical significance was determined at *p* < 0.05. The data were analyzed using GraphPad Prism (La Jolla, CA, USA).

## Data Availability

The data presented in this study are available in this manuscript.

## Supplementary Materials

The following are available online at https://www.mdpi.com/article/10.3390/pathogens10050566/s1, Figure S1: miR-378b gRNA design, Figure S2: Genotyping of mice bred from WT vs. miR-378b heterozygous mice and miR-378b heterozygous vs. miR-378b heterozygous mice., Figure S3. Effect of miR-378b on gross pathology after *Chlamydia* infection. miR-378b^−/−^ (n = 5) female mice were infected with 1 × 10^6^ *C. muridarum*.
