# Supplementary material for: MiR-378b Modulates Chlamydia-Induced Upper Genital Tract Pathology
Source: Pathogens. 2021 May 7;10(5):566. doi: 10.3390/pathogens10050566 (PMC8151610; doi:10.3390/pathogens10050566)
Supplement: Supplementary file 1 [file pathogens-10-00566-s001.zip › pathogens-1193793-supplementary.pdf]

Additional Information

To generate microRNA Knockout Mouse, we utilized CRISPR technology working with Genedits, a Biotech company here at Morehouse School of Medicine. They have had tremendous success in creating many mouse knockout and knock-in strains in the past. They designed and picked guide RNAs (gRNAs) targeting genomic sequences surrounding miR-378b with the highest targeting scores and low off-target potentials. Three gRNAs were selected for each of the miR-378b regions. By using a combination of gRNA 2 and 3, most of miR-378b was deleted; while using gRNA1 and 3, the entire miR-378b was deleted (figure 1).

We *in vitro* transcribed the gRNAs, validated the gRNAs targeting efficiency, and then microinjected mouse zygotes with the CRISPR components, Cas9 mRNA, and gRNAs. Genotyping was performed using PCR with primers flanking the deleted region using genomic DNA from potential founders' tail biopsy when pups were one week old.

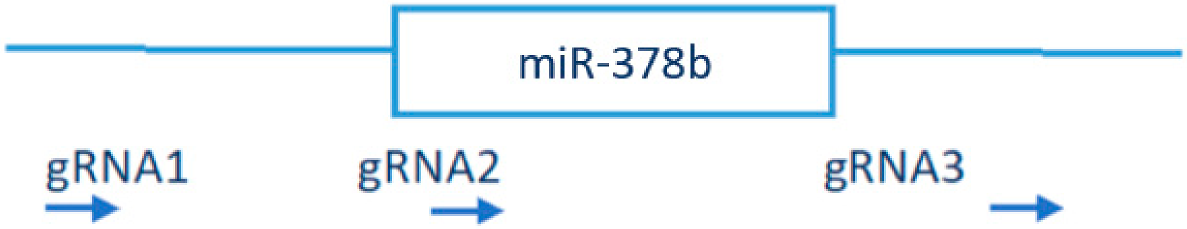

S.Fig.1: miR-378b gRNA design. gRNAs position shown by →

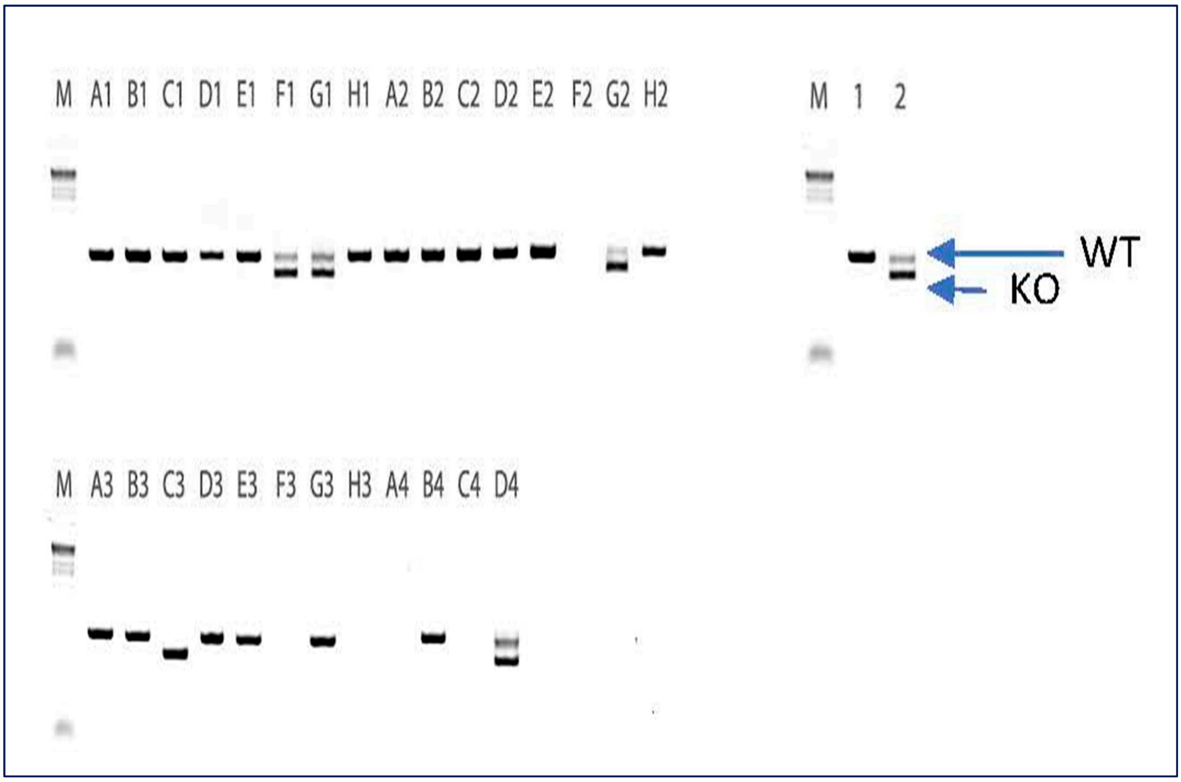

S. Fig. 2: Genotyping of mice bred from WT vs. miR-378b heterozygous mice and miR-378b heterozygous vs. miR-378b heterozygous mice. The figure shows we generated miR-378b heterozygous mice and a miR-378bKO mice. M; molecular weight ladder, 1; WT, 2; heterozygous. WT=443 bp, KO=314 bp.

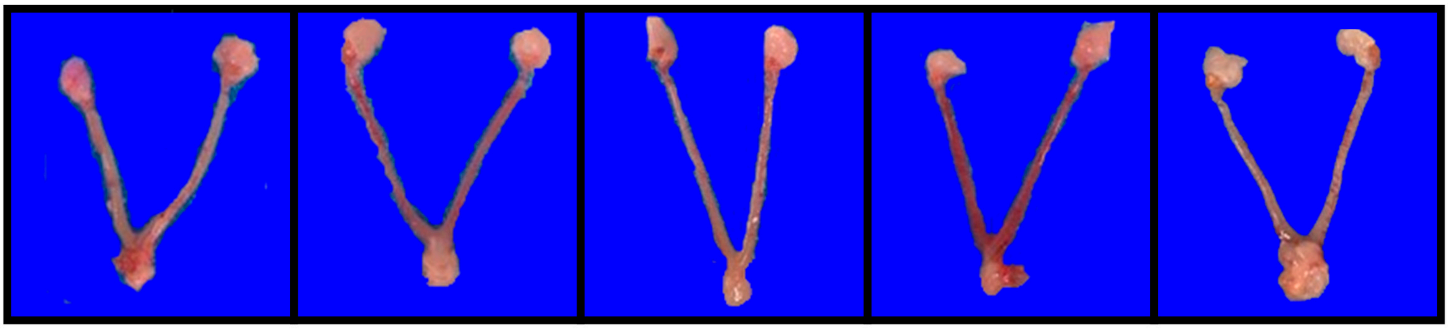

S. Fig. 3: Effect of miR-378b on gross pathology after *Chlamydia* infection. miR-378b$^{-/-}$ (n=5) female mice were infected with $1x10^6$ *C. muridarum*. miR-378b$^{-/-}$ mice had minimal to no uterine tubal pathology.
